# Neurotoxic Effects of Pesticides: Implications for Neurodegenerative and Neurobehavioral Disorders

**DOI:** 10.3390/jox15030083

**Published:** 2025-06-01

**Authors:** Alexandra Andreea Botnaru, Ancuta Lupu, Paula Cristina Morariu, Alexandra Jităreanu, Alin Horatiu Nedelcu, Branco Adrian Morariu, Emil Anton, Maria Luisa Di Gioia, Vasile Valeriu Lupu, Oana Maria Dragostin, Madalina Vieriu, Ionela Daniela Morariu

**Affiliations:** 1 Faculty of Pharmacy, “Grigore T. Popa” University of Medicine and Pharmacy, 700115 Iasi, Romania; botnaru.alexandra@yahoo.com (A.A.B.); jitareanu.alexandra@umfiasi.ro (A.J.); ionela.morariu@umfiasi.ro (I.D.M.); madalina.vieriu@umfiasi.ro (M.V.); 2 Department of Environmental and Food Chemistry, “Grigore T. Popa” University of Medicine and Pharmacy, 700115 Iasi, Romania; 3 Faculty of General Medicine, “Grigore T. Popa” University of Medicine and Pharmacy, 700115 Iasi, Romania; ancuta.ignat1@umfiasi.ro (A.L.); alin.nedelcu@umfiasi.ro (A.H.N.); morariubranco@gmail.com (B.A.M.); emil.anton@umfiasi.ro (E.A.); vasile.lupu@umfiasi.ro (V.V.L.); 4 Department of Pediatrics, “Grigore T. Popa” University of Medicine and Pharmacy, 700115 Iasi, Romania; 5 Department of Internal Medicine, “Grigore T. Popa” University of Medicine and Pharmacy, 700115 Iasi, Romania; 6 Department of Toxicology, Faculty of Pharmacy, “Grigore T. Popa” University of Medicine and Pharmacy, 700115 Iasi, Romania; 7 Department of Morpho-Functional Science I, “Grigore T. Popa” University of Medicine and Pharmacy, 700115 Iasi, Romania; 8 Dipartimento di Farmacia, Salute e Scienze della Nutrizione, Università della Calabria, Arcavacata di Rende, 87036 Cosenza, Italy; ml.digioia@unical.it; 9 Research Centre in the Medical-Pharmaceutical Field, Faculty of Medicine and Pharmacy, “Dunarea de Jos” University of Galati, 800010 Galati, Romania; oana.dragostin@ugal.ro; 10 Department of Analytical Chemistry, “Grigore T. Popa” University of Medicine and Pharmacy, 700115 Iasi, Romania

**Keywords:** pesticide exposure, neurotoxicity, Alzheimer’s disease, Parkinson’s disease, RASFF, neurobehavioral disorder

## Abstract

Pesticides play an essential role in modern agriculture, yet increasing evidence links their widespread use to neurotoxic effects that contribute to both neurodegenerative and neurodevelopmental disorders. In recent years, new classes of pesticides such as neonicotinoids and pyrethroids have garnered attention due to their potential to disrupt neurodevelopment, even at low exposure levels. Furthermore, emerging evidence underscores the involvement of the gut–brain axis, neuroinflammation, and epigenetic modulation in pesticide-induced neuropathology. This review aims to synthesize these latest advancements while highlighting underexplored mechanisms, thereby offering a comprehensive and current perspective on pesticide-related neurotoxicity. Data from the Rapid Alert System for Food and Feed (RASFF) indicate that several food products include residues of pesticides recognized for their neurotoxic properties. Although environmental exposure levels are lower than those in occupational contexts, the magnitude and persistence of food-based exposure demand thorough evaluation. This review integrates evidence coming from epidemiological, in vivo and in vitro investigations, emphasizing the correlations between pesticide exposure and conditions such as Alzheimer’s disease, Parkinson’s disease, and cognitive deficits in children. Neurodevelopmental toxicity is especially alarming since symptoms may manifest subtly and with a delayed onset after early-life exposure, indicating the significant neurotoxic potential of pesticide residues and emphasizing the need for their careful evaluation in food safety assessments. Improved regulatory procedures and public health efforts are essential to reducing long-term brain damage.

## 1. Introduction

In the context of modern agriculture and crop protection, pesticides play a crucial role in controlling harmful organisms. However, their widespread use has raised concerns about their impact on human health and the surrounding environment. Therefore, the utilization of pesticides must be accurately measured and controlled to prevent adverse effects on humans, non-target species, and the ecosystem [1]. The toxicity of pesticides can fluctuate based on their formulation, concentration, and the environmental conditions to which they are subjected [2]. A systematic review of 36 in vivo and in vitro investigations evaluated the toxicity of both active compounds and their related commercial formulations, known as plant protection products (PPPs). Among them, 24 studies indicated heightened toxicity of the formulations relative to the isolated active component, mostly attributable to the inclusion of co-formulants or adjuvants that may augment absorption or induce cellular stress. Specifically, 10 studies examined glyphosate-based herbicides, with 6 determining that Roundup exhibited more toxicity than glyphosate alone, as previously shown in the literature. This highlights the necessity of analyzing the entire pesticide formulation rather than just the active ingredient when evaluating risks to human health and the environment [3,4]. Farmers are mainly exposed to pesticides during mixing, application, and equipment maintenance, primarily through the skin and respiratory tract, with ingestion also possible. Key risk factors include poor safety training, lack of protective gear, and lack of risk perception [5]. Moreover, experts have emphasized the need to improve risk assessment frameworks by incorporating cumulative exposure, mixture toxicity, and biomonitoring data, thereby more accurately reflecting real-world environmental and dietary exposures [6]. Due to developments in risk assessment methodology and the availability of newly acquired toxicological data, an updated cumulative risk assessment is necessary to guarantee that current exposure scenarios and prospective risks are accurately represented [7].

Exposure to various pesticides can affect the development of various neurological disorders, as demonstrated by numerous epidemiological studies [8]. While environmental exposure to pesticides is anticipated to be lower than that in professional contexts, the population of potentially exposed individuals is likely to be greater, encompassing perhaps more vulnerable subgroups such as children, the elderly, those with pre-existing diseases, and those in poor health [9]. The prevalent and excessive use of pesticide combinations has raised concerns over their effects on human health, affecting both the workers involved in their production and application, as well as the pollution of food and water sources [2]. It is essential to identify the correlation between pesticide exposure and the risk of neurological effects [10].

The disparity between the quantity of compounds identified as hazardous to the adult brain and the few recognized as harmful to the more susceptible growing brain is unlikely to diminish in the foreseeable future. This gap arises because toxicity to the adult brain is generally identified following acute poisoning events, which typically exhibit a direct and rapid correlation between the exposure and the harmful consequences, as seen in workplace exposures or suicide attempts. In contrast, developmental neurotoxicity is identified through exposure data from gestation and neurobehavioral data collected 5–10 years postnatally [11].

Neurodegenerative disorders have been extensively researched concerning exposure to neurotoxic pesticides as they disrupt neurotransmission and the functionality of ion channels in the nervous system [10]. These encompass Alzheimer’s disease (AD) and Parkinson’s disease (PD) [12]. Pesticide exposure has been associated with specific neurological effects both in vitro and in vivo, and case reports have demonstrated causal relationships between pesticide exposure and PD and AD [13].

Neurodevelopmental diseases have garnered significant academic interest over the past few decades, particularly regarding possible environmental factors that might affect brain development and function [14]. Official statistics indicate a rise in the frequency of neurodevelopmental disorders [15]. Chronic exposure to pesticides and persistent organic pollutants may disrupt thyroid homeostasis in both mothers and infants, potentially impacting neurodevelopment and behavior in embryos and children [15]. Since concerns regarding the cognitive consequences of pesticides emerged, several research teams have endeavored to undertake tests to explore this correlation and clarify the underlying processes [16]. Concerns regarding developmental neurotoxicity from pesticides have been heightened by new epidemiological findings indicating that children exposed prenatally or throughout early postnatal life experience numerous neurological impairments [17]. Developing fetuses and children are at significant risk of exposure and associated harmful effects due to an underdeveloped blood–brain barrier and detoxifying mechanisms. The use of pesticide mixes in real-life scenarios is frequently noted and correlates with an increased occurrence of pesticide-related poisonings and fatalities [18]. However, the degree of risk can vary considerably depending on the world region, as factors such as regulatory policies, pesticide usage patterns, and public health infrastructure differ widely. Pesticide poisoning is recognized as a significant public health issue in countries with low or middle incomes [19].

## 2. RASFF Insights

The general population is mostly exposed to pesticide residues through the consumption of food products treated with these chemicals. The assessment of dietary intake of food-based pesticides may be conducted for certain demographic groups, including children, teens, and seniors, taking into account both chronic and acute exposure, and may be integrated with water and home exposure evaluations [10].

To safeguard human health and facilitate international trade, the European Union and the Codex Alimentarius Commission have set MRLs (maximum residue limits) for pesticide residues in food products. The objective is to establish MRL values that minimize pesticide residues in food to the lowest practical and safe levels for consumers, following prudent application to safeguard crops [20].

MRLs and Health-Based Guidance Values (HBGVs) fulfill distinct yet complementary functions in risk assessment and food safety regulation. HBGVs, including the Acceptable Daily Intake (ADI) and the Tolerable Upper Intake Level (UL), are scientifically established metrics that delineate the quantity of a chemical that can be ingested daily over a lifetime without presenting a significant health risk. These values derive from toxicological data and are utilized to evaluate comprehensive dietary exposure from all sources. Conversely, MRLs are regulatory thresholds established for the presence of residues from chemicals like pesticides or veterinary pharmaceuticals in food items. Their objective is not to signify safety directly but to guarantee that the presence of such residues in food does not result in consumer exposures surpassing the relevant HBGVs. Consequently, HBGVs serve as toxicological reference points, whereas MRLs work as regulatory instruments intended to maintain real exposure within acceptable limits [21].

If proper agricultural and manufacturing practices are rigorously enforced, pesticide residues will be reduced to below the MRLs [22]. Given the low-level MRLs established by regulatory organizations, the development of efficient, precise, and sensitive analytical methodologies is imperative [23]. Instruments for transnational communication of food safety hazards, such as the European RASFF, are becoming progressively vital for consumer protection across global supply chains [24]. The RASFF Window features an RASFF consumer portal that offers information on recent food recalls and public health alerts in member nations associated with RASFF notifications. Notifications from RASFF, submitted by a national food safety authority, are validated by the European Commission, which supervises the system, and thereafter distributed to the contact points of network members. Impacted products can then be traced and appropriate actions implemented [24].

The MRL value is calculated using statistical methods and is typically set to encompass at least the upper confidence interval of the 95th percentile of the anticipated residue distribution. Consequently, an estimated 1% of MRL exceedances are anticipated, even with full adherence to Good Agricultural Practices (GAPs). According to European Commission statement from 2021, a sample is considered non-compliant when at least one pesticide is detected at a level such that, after considering measurement uncertainty, the lower bound of the distribution exceeds the MRL value Following identification of a non-compliant sample, it is imperative to initiate action at the Member State level in accordance with Article 50 of Regulation (EC) No 178/200260. Typically, Member States respond to non-compliance with suitable measures, such as administrative penalties, RASFF notices, and further steps [25]. In addition, we accessed the RASFF Window on 30 December 2024 and investigated the alerts related to pesticide residue hazards in food products using the search interval from 1 January 2022 to 30 December 2024. Combining these search filters yielded a total of 2889 alerts [26]. We exported the data to Microsoft Excel, where it was further processed to produce descriptive statistics. Variables such as product category, type of pesticide, country of notification, and year of report were grouped and described in terms of frequency and proportion. It is important to note that the RASFF system already categorizes food products into standardized groups, which were used directly in the analysis. Data processing steps included filtering, sorting, and grouping based on the selected variables, followed by visualization through charts generated in Microsoft Excel. No inferential statistical tests were performed, as the goal was to offer a narrative overview of the data and highlight observable trends across the reporting period.

**Figure 1:**
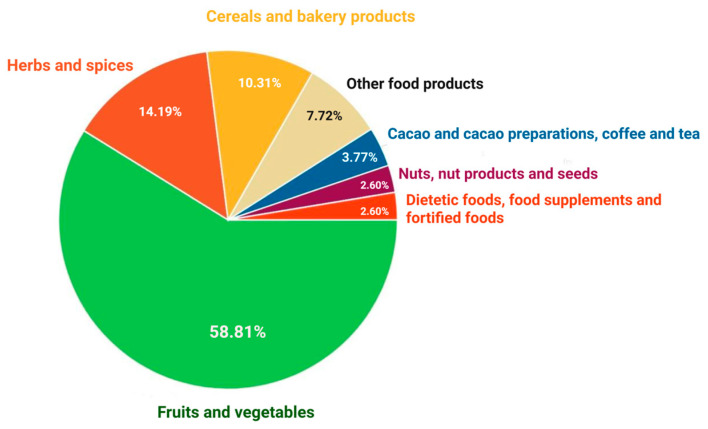
Distribution of pesticide residue hazards by food product category.

The chart from Figure 1 illustrates the percentage distribution of pesticide hazards across several categories of food products. The highest percentage, 58.81%, corresponds to fruits and vegetables, indicating that those products are the most susceptible to pesticide hazards, perhaps due to the extensive application of phytosanitary treatments in agriculture. Herbs and spices, accounting for 14.19%, may also acquire residues from cultivation and handling operations. Cereals and bakery products constitute 10.31% of total hazards related to pesticide residues, signifying moderate pesticide exposure, likely resulting from treatments administered to cereals prior to harvest or during storage. The remaining categories, including cocoa and cocoa preparations, coffee and tea (3.77%), dietetic items and food supplements (2.60%), nuts and seeds (2.60%), and other food products (poultry meat and poultry meat products, fats and oils, food additives and flavorings, prepared dishes and snacks, fish and fish products, soups, broths, sauces and condiments, non-alcoholic beverages, honey and royal jelly, confectionery, feed materials, ices and desserts, meat and meat products, milk and milk products, plant protection products and other food products/mixed) exhibit a lower percentage.

**Figure 2:**
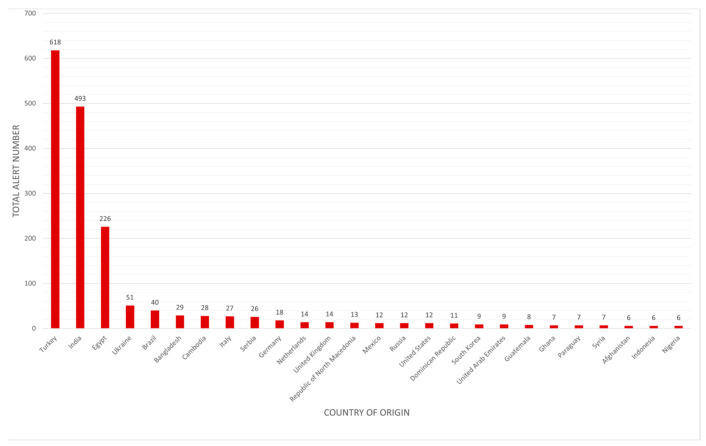
Number of pesticide residue alerts by country of origin.

Figure 2 illustrates the distribution of hazards related to pesticide residue alerts according to the country of origin of the foods in which problems have been identified. This shows notable disparities in pesticide management among the countries of origin. Based on a total of 2889 notifications, Turkey is the country with the greatest number of food alerts related to pesticides with 618 alerts, accounting for about 21.4% of all alerts. Countries ranked highest, including Turkey, India, and Egypt, should be prioritized for the enforcement of more stringent procedures to regulate and verify pesticide levels in food products.

**Figure 3:**
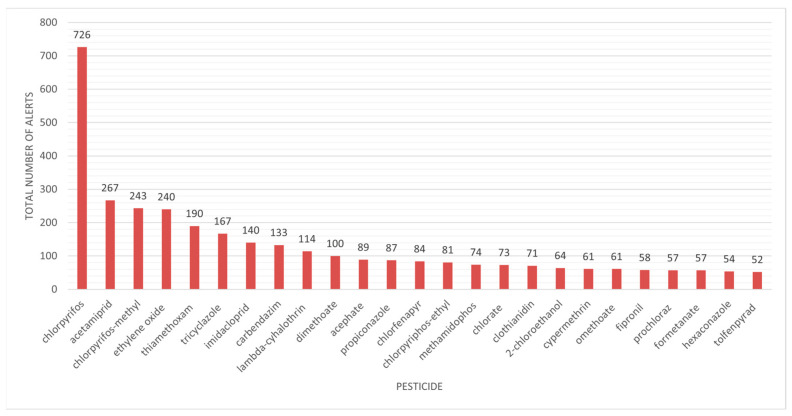
Pesticide residues in food products linked to alerts in the RASFF Window database.

We aimed to determine which pesticide type was more frequently associated with the generated alerts. Chlorpyrifos generated 726 notifications, followed by acetamiprid with 267 notifications and chlorpyrifos-methyl with 243 notifications, far surpassing other chemicals (Figure 3). The major problem is that the use of this pesticide is unauthorized. Chlorpyrifos (O,O-diethyl-O-3,5,6-trichloro-2-pyridyl phosphorothioate), an organophosphate pesticide, is essential for pest management across many crops, fruits, and vegetables. It is a recognized acetylcholinesterase (AChE) inhibitor that leads to the accumulation of acetylcholine, resulting in overstimulation of postsynaptic receptors and resulting negative consequences [27]. Chlorpyrifos and other organophosphate exposures in the USA caused projected yearly losses of up to billions of dollars due to cognitive declines and abnormalities in brain development. As a result, the European Union prohibited the usage of chlorpyrifos in 2020, followed by the US in 2021 [28].

Despite the recommendation leading to the ban of the organophosphate pesticide chlorpyrifos in Europe since 2020, chlorpyrifos remains prevalent in other continents. Furthermore, the global import and export market for fruits and vegetables ensures its continued presence in the global food chain [15].

Multiple factors may contribute to MRL breaches. Regarding samples originating from third countries, the key factors are the use of unapproved pesticides—for which no import tolerance is provided—(chlorpyrifos and thiamethoxam in cumin seed and rice from India; imidacloprid in rice from India and Pakistan), misuses of non-approved pesticides (chlorpyrifos-methyl in grapefruit from Turkey and imidacloprid in dragon fruit from Thailand), non-compliance with GAP (acetamiprid and lambda-cyhalothrin in teas from China), and pollution resulting from prior pesticide application (absorption of residues from the soil). Regarding the samples from the national market (reporting countries), the main reasons for exceeding MRLs are the application of authorized pesticides not designated for the crop specified in the GAP authorization (imazalil in potatoes, pirimiphos-methyl in tomatoes, and fosetyl in barley grain), natural occurrence of the chemical in the crop (copper compounds in buckwheat and other pseudocereals), cross-contamination (acetamiprid and prosulfocarb in kale), environmental contamination by persistent organic pollutants (dieldrin in carrots or squash) and environmental pollution, the existence of biocide residues previously utilized as pesticides, which are still subject to monitoring under pesticide regulations (chlorate), and utilization of pesticides not approved by the EU (acrinathrin in spinach, chlorpyrifos in Chinese cabbage and barley grain, chlorpyrifos-methyl in grapefruits, and chlorfenapyr in tomatoes) [25].

The chart data show the incidence of RASFF warnings regarding diverse pesticide residues found in food, clearly indicating that several of the most commonly reported chemicals are recognized or presumed neurotoxicants. Chlorpyrifos, with 726 notifications, significantly surpasses others, indicating its extensive application and recognized neurotoxicity, especially regarding neurodevelopmental impacts. Other pesticides with numerous notifications, including acetamiprid, chlorpyrifos-methyl, thiamethoxam, and imidacloprid, are predominantly neonicotinoids or organophosphates, substances that affect the nervous system and have been associated with detrimental impacts on cognitive and behavioral development, particularly during early life stages. Substances with fewer alerts, such as fipronil or methamidophos, are nonetheless linked to considerable neurotoxic risk. The prevalence of these substances in food-related warnings highlights a significant issue: food is not just a secondary exposure pathway but may serve as a persistent, low-dose source of neurotoxicants for the general populace. This trend underscores the necessity for comprehensive risk assessment methodologies that account for both acute toxicity and chronic neurotoxic impacts, especially for at-risk populations like children and the elderly. Moreover, these data underline the necessity of rigorous surveillance of pesticide residues in order to prevent food fraud, particularly for commonly consumed fresh produce like fruits and vegetables.

## 3. Neurotoxicity of Pesticides

There is substantial evidence that organophosphate pesticides (OPPs), carbamate pesticides, pyrethroids, and organochlorinated (OCPs) insecticides induce acute neurotoxicity. Numerous epidemiological studies have identified an increased risk of behavioral and/or neurological long-term consequences in individuals exposed to pesticides over prolonged periods [29]. In conditions of poisoning, prompt administration of an anticholinergic agent (atropine) counteracts cholinergic hyper-activation at muscarinic receptor sites, whereas oximes (obidoxime/pralidoxime) serve to restart blocked enzymes. While enhancing survival rates, the combination of atropine and oxime is inadequate to avert significant cerebral impairment following OPP poisoning, particularly if administered late [30]. Experimental evidence from research investigations indicates that many pesticides induce mitochondrial dysfunction and oxidative stress across several cell types and animal models, constituting a principal mechanism of pesticide harm [31].

### 3.1. OPPs

The primary mechanism of OPP toxicity is the inhibition of acetylcholinesterase in the nervous system, leading to excessive cholinergic activity due to the buildup of acetylcholine in synapses and neuromuscular junctions. They have been associated with a higher incidence of chronic deleterious effects compared to carbamates, including organophosphate-induced delayed polyneuropathy, cognitive sequelae, and developmental neurotoxicity [32,33]. Seven to fourteen days following acute intoxication, individuals exhibit distal axonal degradation accompanied by later secondary demyelination impacting peripheral neurons and both ascending and descending pathways within the spinal cord [34]. Organophosphate-induced delayed polyneuropathy frequently occurs after exposure to organophosphate compounds that have low anticholinesterase activity, such as triorthocresyl phosphate. Nonetheless, after exposure to the currently available organophosphate compounds with potent anticholinesterase action, it is notably rare. Experimental research on hens has identified many neuropathic organophosphates, including mipafox, merphos, leptophos, cyanophos, and trichloronat [35,36]. The adult hen is the preferred animal model for examining the toxicity pathways linked to organophosphate-induced delayed neuropathy, as it consistently develops histological lesions and clinical manifestations, such as ataxia and paralysis, following a single dose of neuropathic organophosphates [37].

OPPs can affect various areas of the brain, particularly the hippocampus, which is the primary region responsible for memory and learning regulation. The hydrophobic character of OPPs contributes to its accumulation in adipose tissue, perhaps leading to intermediate syndrome with delayed neurological damage due to the production of oxidative stress [38,39]. Consequently, the probability of delayed and sustained AChE inhibition increases with OPPs [40]. Furthermore, pharmacokinetic investigations in animals have revealed that OPPs with high lipophilicity, such as chlorpyrifos, may be identified in adipose tissue for prolonged durations and can be released in milk or during lipid metabolism. Maternal milk is considered an ideal carrier for biomonitoring exposure to lipophilic pollutants in both mothers and newborns due to its abundance, non-invasive sampling capability, and high lipid content [41]. Residues of chlorpyrifos have been detected in the breast milk generated by breastfeeding women, suggesting that stored organophosphates in adipose tissue are mobilized into milk lipids [42,43]. In obese patients, the concentration of organophosphate in the vascular compartment persists for a longer time. Consequently, obese people exposed to OPPs from the highly lipophilic category experience prolonged toxicity [44]. The levels of lipophilicity and the patient’s adipose tissue may influence the prognosis following poisoning. A Korean study investigated the results of 112 severely poisoned patients, among whom 40 individuals were obese. Individuals with a body mass index over 25 underwent prolonged mechanical breathing, longer intensive care unit stays, and a higher overall duration of hospital hospitalization [44]. However, biomonitoring studies and analytical techniques for evaluating these chemicals in human adipose tissue are limited [39,40].

**Figure 4:**
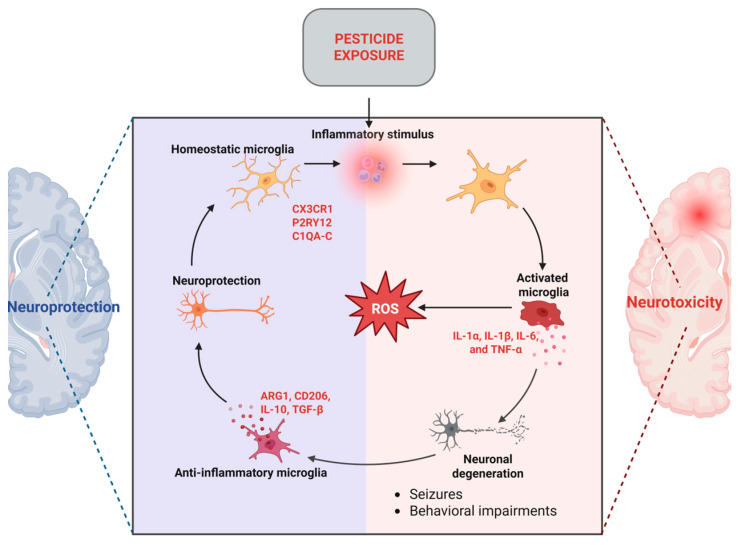
Microglia-mediated mechanisms in neuroinflammation (created with Biorender.com) https://app.biorender.com/illustrations/682f39cb10a1f38bf3f8782a (accessed on 26 May 2025). ROS = Reactive Oxygen Species.

Compelling data indicate that inflammatory responses contribute to organophosphate-mediated neuronal damage, resulting in unfavorable neurological prognoses. A substantial portion of this information has been obtained from experimental models of OPP poisoning, which is known to induce proinflammatory responses, ultimately resulting in delayed neuronal damage and neurological impairments. One of the initial events linked to OPP intoxication is the activation of microglia. Activated microglia have been demonstrated to elicit inflammatory cytokines, including IL-1α, IL-1β, IL-6, and TNF-α, in mouse brains subsequent to organophosphate overdose [45,46]. Neuroinflammatory reactions were seen to precede neurodegeneration, seizures, and substantial behavioral impairments (Figure 4). Nonetheless, the underlying relationship between neuroinflammation and organophosphate-induced neurological impairments remains little understood [45].

Even minimal exposure to OPPs during early development, at dosages insufficient to induce acetylcholinesterase inhibition, can adversely affect neurobehavioral and cognitive function, frequently evident later in childhood. Neurotoxic effects may result from the targeting of diverse brain cells. Early identification of neurotoxicity can be accomplished by detecting particular proteins generated after neuronal or glial damage in blood or serum. These proteins are vital constituents of neurons, encompassing the axon, dendrites, synapses, soma, and myelin sheath [47]. Moreover, recent findings elucidated a novel mechanism of synaptic dysfunction common to various classes of pollutants, specifically impaired hippocampal mGluR-LTD, which could connect molecular alterations in protein signaling with cognitive and behavioral deficits [48].

### 3.2. OCPs

OCPs comprise a varied array of agents categorized into three specific chemical classes: dichlorodiphenylethane, chlorinated cyclodienes, and chlorinated benzenes. OCs serve as efficient insecticides owing to their low volatility, chemical stability, lipid solubility, and slow biotransformation and degradation rates. Consequently, these chemicals exhibit significant persistence and biomagnification, rendering them a substantial threat. Numerous studies have associated exposure to OCs with an elevated risk of neurodegeneration, as they disrupt the neurological system and result in uncontrolled neuronal stimulation [3,49]. DDT was the first contemporary synthetic pesticide developed. The primary route of exposure is through the ingestion of contaminated food, predominantly found in meat and dairy products. Due to the progressive degradation of the parent compound, organochlorines can build up in adipose tissue and breast milk. In light of these concerns, DDT was banned for agricultural use in the United States in 1972. Other countries likewise restricted or curtailed its use in agriculture during the 1970s and 1980s. It remains employed for agricultural purposes in several countries, particularly in poorer nations. Various OCPs, including DDT, aldrin, chlordane, and heptachlor, are considered persistent environmental pollutants [3].

### 3.3. Carbamates

Carbamate insecticides originate from carbamic acid and exhibit varying levels of acute oral toxicity, ranging from moderate to low toxicity (carbaryl) to extremely high toxicity (aldicarb; LD_50_ = 0.8 mg/kg). The toxicological mechanism of carbamates parallels that of OPPs, since both inhibit AChE [50]. The link between carbamate and AChE is far less stable than that created with an OPP, leading to spontaneous decarbamylation by gradual hydrolysis, which ultimately reactivates AChE. Furthermore, the enzyme–inhibitor combination does not experience the aging process characteristic of OPPs. Nevertheless, the temporary inhibition of AChE by a carbamate facilitates the accumulation of acetylcholine at muscarinic and nicotinic synapses within the sympathetic and parasympathetic systems, as well as at neuromuscular junctions of both striated and smooth muscles, ultimately leading to a clinical cholinergic crisis [51]. The Environmental Protection Agency has categorized aldicarb in the highest hazard category and established stringent regulations for its distribution and application [52].

### 3.4. Pyrethroids

Pyrethrins are natural insecticides extracted from the chrysanthemum flower. Pyrethroid insecticides are synthetic analogs of pyrethrins, engineered for enhanced stability. With the introduction of more restrictions on OPPs, the utilization of pyrethroid insecticides has escalated [3]. They can be classified into two groups based on their chemical structures, toxicity profiles, and target locations. Type I pyrethroids are classified as non-cyanopyrethroids (they lack a cyano group) and inactivate the sodium channels in a very short duration. Type II pyrethroids include an alpha-cyano group. The persistent depolarization of the neuronal membrane causes the alpha-cyano group to maintain prolonged sodium channel activation [53].

Research on pyrethroid neurotoxicity first concentrated on acute neurotoxicity and the characterization of distinct syndromes induced by various pyrethroid insecticides. Recent studies have concentrated on the possible neurotoxicity associated with prolonged, low-level pyrethroid exposures that do not lead to overt intoxication in people and animals. Despite the perception that the general population’s exposure to pyrethroids is minimal, humans possess an absence of serum carboxylesterases, which are essential for the hydrolytic detoxification of pyrethroids. This may result in a diminished ability for humans to digest pyrethroids [31].

Recent findings from HBM4EU (Human Biomonitoring)-aligned studies (2014–2021) have yielded significant biomonitoring data regarding pyrethroid exposure among various European populations, encompassing both children and adults. Exposure to pyrethroids was predominantly evaluated by urine metabolites, with 3-phenoxybenzoic acid (3-PBA) serving as a commonly used marker for cumulative exposure. Although substance-specific evaluations indicated limited risk for health, the 95th percentile concentrations of 3-PBA were above conservative screening thresholds derived from HBGVs in children across all assessed demographics. To address this issue, researchers employed a tiered risk assessment methodology that converts the intricacies of pyrethroid application and metabolism into a systematic framework for assessing cumulative risk. These findings underscore the need of ongoing biomonitoring and the necessity for adaptable risk assessment methods, particularly for safeguarding vulnerable subpopulations like children [54].

Permethrin, deltamethrin, and cypermethrin are the three most often used chemicals [3]. A recent physiologically based pharmacokinetic study indicated that exposure to the Type II pyrethroid deltamethrin will lead to a twofold increase in peak brain concentration in humans compared to rats. Neurological consequences, such as cognitive impairment, have been noted after pyrethroid exposure among pesticide applicators and their families [31]. Deltamethrin disrupts voltage-gated sodium channels (VGSCs) and impairs the proper operation of calcium channels and glutamatergic receptor activation, while also affecting both GABAA and nicotinic acetylcholine receptors, eventually resulting in a convulsive state [55]. Cypermethrin, a fourth-generation pyrethroid insecticide, is extensively utilized globally to manage cotton pests and ectoparasites such as ticks and mites in livestock. Cypermethrin has been recognized as a serious pesticide associated with human health hazards. [53] Multiple studies have been conducted to elucidate the toxicity of cypermethrin, especially regarding its impact on neurological structures. They function by engaging with the nerve cell membrane and inhibiting the closure of the sodium channel’s ion gates during repolarization. The nervous system’s capacity to transfer signals is significantly impaired, leading to spontaneous membrane depolarization or repeated discharges [53].

### 3.5. Neonicotinoids

Currently, there are seven principal neonicotinoid pesticides utilized globally: Thiacloprid, Dinotefuran, Acetamiprid, Clothianidin, Thiamethoxam, and Imidacloprid. Neonicotinoids selectively affect the nicotinic acetylcholine receptors (nAChRs) within the neurological systems of insects. [56]. Certain compounds (clothianidin, thiamethoxam, imidacloprid) are prohibited in the EU because of their impact on pollinator populations. Moreover, neonicotinoids have been documented to inadequately traverse the blood–brain barrier, with certain effects observed in the brains of mice and zebrafish. Neonicotinoids and their metabolites can influence neurodevelopment and neurotransmission, as well as generate oxidative stress and neuroinflammation in rodent models. Evidence indicates that acetamiprid accumulates in the brain following prolonged oral dosing in adult mice, affecting nAChR expression levels without inducing significant histomorphological alterations in the brain [57]. Thiacloprid-induced toxicity, demonstrated via both in vivo and in vitro investigations, has been linked to neurotoxicity, hepatotoxicity, nephrotoxicity, and detrimental effects on several organs and systems. Exposure to thiacloprid has been linked to a significant increase in oxidative stress in the brain, resulting in adverse consequences on neuronal health. This is demonstrated by a significant reduction in total antioxidant capacity (TAC) alongside an increase in malondialdehyde (MDA), protein carbonyls (PCs), and reactive oxygen species (ROS). These oxidative stress indicators signify considerable cellular damage, leading to neurotoxicity. The heightened oxidative stress caused by thiacloprid is exacerbated by its ability to produce DNA damage, as seen by enhanced comet test endpoints, which serve as a marker for oxidative DNA damage. The detected oxidative damage acts as a catalyst for apoptotic pathways, initiating a series of events that eventually undermine cellular integrity [56].

Several studies indicate the presence of neonicotinoid residue mixes in food and the environment. Chen et al. demonstrated that 72% of examined fruits, 45% of vegetables, and 50% of honey had a minimum of two distinct neonicotinoids in a single sample. The presence of neonicotinoids in food has heightened interest in the cumulative toxicity of combination pesticides. Low doses of neonicotinoids can activate the nervous system, whereas higher amounts may lead to receptor blockade, paralysis, and mortality [2]. Fatalities due to neonicotinoid poisoning are predominantly attributed to imidacloprid; yet, the mortality rate linked to imidacloprid remains low, varying from 0% to 4.2%. Occasional fatalities due to acute imidacloprid intoxication have also been documented in the literature [58].

## 4. Neurodegenerative Disease

### 4.1. Parkinson’s Disease

Parkinson’s disease (PD) is a prevalent neurological disease that results in considerable morbidity and deterioration in quality of life. The condition arises from the degeneration of dopaminergic neurons in the substantia nigra pars compacta, with oxidative stress being a significant element in the course of the illness [59]. The symptoms involve bradykinesia, rigidity of the muscles, resting tremor, and impairments in posture and walking. Non-motor characteristics encompass olfactory impairment, cognitive deficits, psychiatric manifestations, sleep disturbances, autonomic dysfunction, pain, and fatigue [60]. The complex nature of this pathology presents clinical obstacles, such as the inability to achieve a definitive diagnosis in the first stages and difficulties in symptom treatment during advanced stages [60]. Despite extensive study and development in molecular pathophysiology and pharmacology, no definitive therapy strategy exists for the management of PD [10].

Solid data indicate that the prevalence of PD is significantly linked to exposure to environmental pollutants [61]. Various environmental exposures have been investigated in relation to PD risk, including tobacco use, pesticide exposure, and traumatic brain injury [62]. A 2018 meta-analysis revealed that extended pesticide exposure correlated with an increased risk of PD by up to 11% and a 2019 meta-analysis shows that pesticide exposure is associated with a 50% increased risk of neurodegenerative diseases such as amyotrophic lateral sclerosis and PD [63]. Additionally, a 2017 meta-analysis of case–control studies found that pesticides are linked to a heightened risk for PD and modifications in genes associated with the disease [64].

Rural life and agricultural employment are considered major risk factors for developing PD, mostly owing to heightened exposure to pesticides. Residing in metropolitan environments characterized by elevated air pollution levels has been associated with a heightened risk for getting PD. Tanner et al. indicated that the risk of parkinsonism escalated with the use of pesticides such as rotenone, dieldrin, and paraquat, a conclusion corroborated by many investigations [62]. In France, PD is regarded as an occupational disease among farmers that utilize pesticides [59]. A screening test including 400 farmers for neurological illness and occupational pesticide exposure has indicated an elevated risk of PD with an unidentified causal mechanism [29]. Epidemiological evidence indicated a significant correlation between occupational pesticide exposure and neurodegeneration, whereas genetic and biochemical research has associated the ubiquitin–proteasome system (UPS) with the etiology of PD (e.g., dieldrin, paraquat, rotenone, maneb, and ziram) [10].

After acute OPP poisoning, let-7g was seen to be downregulated, a pattern previously noted in the cerebrospinal fluid of PD patients. Furthermore, miR-141 was identified as downregulated (miR-141–5p) following acute OPP poisoning and was reported as downregulated (miR-141−3p) in neuronal samples from PD patients. MiR-126 was identified as elevated in the blood of individuals exposed to OPPs and in two investigations using dopaminergic neuron samples from PD patients [65].

Pesticides and herbicides have been more extensively investigated than other pollutants because of the structural similarities between paraquat (herbicide) and rotenone (insecticide) and the established parkinsonism-inducing metabolite 1-methyl-4-phenylpyridinium (MPP+) [62]. Both in vivo and in vitro investigations have shown that paraquat treatment might result in neuronal cell death. Multiple epidemiological studies have demonstrated that prolonged exposure to paraquat leads to nervous system abnormalities, including a decrease in dopamine neurons in the substantia nigra, possibly resulting in PD [66]. Pesticides accelerate the production of alpha-synuclein fibrils. Moreover, rotenone, paraquat, and dieldrin induce a conformational alteration in alpha-synuclein, hence accelerating its fibrillization and contributing to PD [13]. Exposure to paraquat and maneb has been demonstrated to elevate the risk of PD by 200% [45]. An increasing number of research suggests that stress-activated kinases, such as c-Jun N-terminal kinase (JNK) and p38 kinase, are pivotal in the degenerative process triggered by paraquat. Sequential phosphorylation of JNK, alongside the activation of caspase-3 and p53 transcription factors, has been documented in animal and in vitro models of PD with paraquat [67].

**Figure 5:**
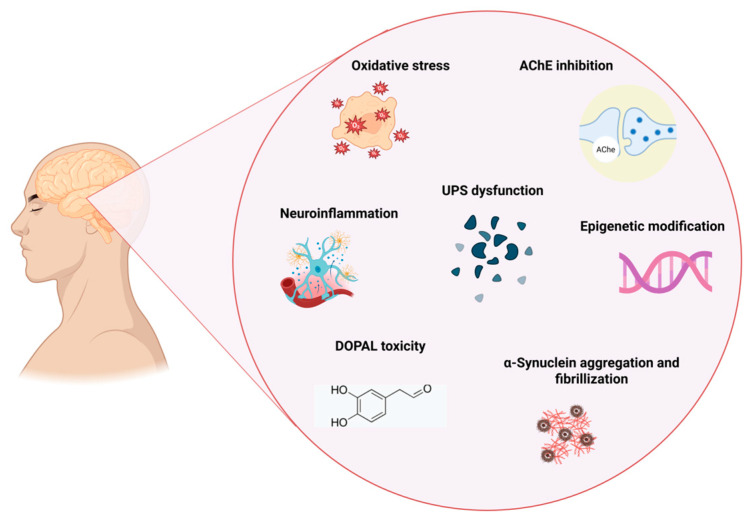
Mechanism linking pesticide exposure to PD (created with Biorender.com) https://app.biorender.com/illustrations/67d6d93587a81252a93927ba (accessed on 1 May 2025). AChE = Acetylcholinesterase. UPS = Ubiquitin–Proteasome System. DOPAL = 3,4-dihydroxyphenylacetaldehyde.

Oxidative stress has been demonstrated to significantly contribute to the development of chronic and neurodegenerative disorders, including PD, and hence, pesticides may induce these neurotoxic processes through oxidative stress [59]. Oxidative stress results from an imbalance between free radical production and the body’s capacity to mitigate damaging consequences through antioxidants. Cell-damaging free radicals include hydrogen peroxide, the hydroxyl radical, nitric oxide (NO), and the superoxide radical, whilst antioxidants comprise superoxide dismutase (SOD), catalase, glutathione, and uric acid. Oxidative stress and mitochondrial malfunction have been demonstrated to contribute to the sequence of events resulting in dopaminergic neurodegeneration [45]. Several studies indicate that paraquat adversely affects the developing neurological system through the production of reactive oxygen species, such as superoxide [66]. Biomarkers of oxidative stress, including glutathione-S-transferase (GST) and total antioxidant capacity, as well as genotoxicity indicators like the comet assay, are significantly affected by pesticides [59]. As illustrated in Figure 5, pesticide exposure contributes to PD through multiple mechanisms, including oxidative stress, AChE inhibition, neuroinflammation, and epigenetic modification, ultimately leading to dopaminergic neuron degeneration.

Previous studies have connected pesticide exposure and genes related to lysosomal function with an increased risk of PD. Therefore, a group of researchers assessed the prevalence of variations in lysosomal function genes among participants of the Parkinson’s, Environment, and Genes (PEG) study exposed to ambient pesticides from agricultural sources. A total of 757 PD patients, predominantly of White European/non-Hispanic descent (75%), were evaluated for genetic variations across 85 genes utilizing a bespoke amplicon panel. The results indicate that gene variants linked to lysosomal function, particularly autophagy, were prevalent in PD patients exposed to agricultural pesticides, implying that compromised lysosomal function may create a predisposition to developing PD in the context of pesticide exposure [68].

Several studies have examined genome-wide DNA methylation patterns in PD concerning pesticide exposure. Paul et al. analyzed DNA methylation in whole blood samples from PD individuals (n = 342) and control subjects (n = 238), identifying 70 differentially methylated CpGs (cytosine–guanine sites), 27 of which were linked to OPP exposure. DNA methylation changes linked with exposure were seen in both PD patients and controls. In comparison to blood, just 14 differentially methylated CpG sites linked to OPP exposure were detected in saliva (n = 128 cases, n = 131 controls) [69]. Similar research characterized sex-stratified genome-wide blood DNA methylation patterns, SNP (single-nucleotide polymorphisms) genotypes, and pesticide exposure among agricultural workers (71 early-stage PD cases, 147 controls) and investigated replication in three separate samples with diverse demographics. Employing a region-based methodology, they identified a greater number of connections between blood DNA methylation and PD in females (69 regions) compared to males (2 regions). In 48 female subjects, models incorporating genotype or both genotype and pesticide exposure significantly enhanced the explanation of interindividual variance in DNA methylation, and considering these factors reduced the anticipated impact of PD on DNA methylation. The findings indicated that genotype, along with genotype–exposure interactions to a lesser extent, contributed to the variability in PD-associated DNA methylation [70]. Go et al. detected seven differentially methylated sites (DMPs) in occipital brain samples from PD cases with over ten years of exposure to plantation work, in comparison to unexposed individuals. The two most identified DMPs were associated with PTGDS (Prostaglandin D2 Synthase) and PEX19 (Peroxisomal Prostaglandin D2 Synthase Biogenesis Factor 19), exhibiting increased DNA methylation in plantation workers. A total of 123 DMPs were discovered in the blood samples, with the two most significant DMPs associated with WNT16 and ENTPD8, although they only achieved a suggestive significance level (*p* < 0.0001) [71].

In addition to genetic susceptibility related to lysosomal function, another key mechanism implicated in the development of PD following pesticide exposure is neuroinflammation. Neuroinflammation, the inflammation of the central nervous system, is regarded as a defining characteristic of several neurological disorders, including PD [45]. While it remains uncertain if neuroinflammation precipitates pesticide-induced neurodegeneration, accumulating data indicate that an inability to mitigate neuroinflammatory processes may contribute to heightened susceptibility to pesticide neurotoxicity. Moreover, new research provides further data that redirect attention from a neuron-centric perspective to glial-associated neurodegeneration subsequent to pesticide exposure [45]. Microglia are the principal immune cells of the central nervous system and exhibit significant similarity to peripheral macrophages. Microglia are activated upon acute or chronic exposure to certain substances or triggers. Activated microglia secrete several pro-inflammatory cytokines, triggering an inflammatory response that disrupts normal brain function and eventually exacerbates disease development and severity [72]. Studies conducted on laboratory animals and cell cultures indicate that pesticides trigger microglial activation, as shown in Table 1. This activation plays a crucial role in neuroinflammation, contributing to the progression of neurodegenerative diseases like PD. Table 1 presents a selection of in vivo and in vitro studies that demonstrate the neurotoxic effects of various pesticides, highlighting their potential role in the pathogenesis of PD through mechanisms such as dopaminergic neuron degeneration and oxidative stress.

Benomyl (fungicide) is extensively utilized in India for agricultural crop development. Research on benomyl indicated that prolonged exposure may result in the suppression of the enzyme aldehyde dehydrogenase, leading to DOPAL (3,4-dihydroxyphenylacetaldehyde) toxicity. DOPAL is extremely harmful to dopamine neurons, and continued exposure results in dopamine degeneration and PD [73].

**Table 1:** Experimental evidence from animal and cell models linking pesticide exposure to parkinsonian neurodegeneration.

Pesticide	Experimental Model	Outcome	Reference
Paraquat Maneb	Mice	Microglial activation Neuroinflammation + neurodegeneration—NADPH oxidase activation	[61]
Paraquat	Murine microglial line BV2 cells	Microglial activation ↑ Pro-inflammatory cytokines Neuroinflammation—HSP60/TLR4 signaling	[74]
Paraquat	Mice (PD)	Postmortem brain samples—↑ astrocytic senescence Elimination of senescent cells Neurodegeneration	[75]
Paraquat	Neuro-2a cells	(−) Cell proliferation Induced cell death	[66]
Paraquat	SH-SY5Y cells	(−) Cell viability Mitochondrial damage Cell apoptosis in A549 cells	[76]
Paraquat	Mice (*n* = 10)	(−) Proliferation of NPCs in hippocampus Changes in cell differentiation pathways	[66]
Paraquat	Zebrafish (*n* = 24)	Mitochondrial dysfunction Upregulation of stress-related genes	[77]
Azamethiphos Chlorpyrifos Dieldrin Heptachlor	Rats Phaeochromocytoma cell line PC-12 (Total = 2000 cells)	Developmental neurotoxicity—neurite outgrowth, transcriptional changes Neurite outgrowth—induction of gap-43	[78]
Rotenone	Rats (PD)	MicroRNA dysregulation Loss of TH (+) cells Microgliosis	[79]
Rotenone Tebufenpyrad	CRL-2690 cells Rats	Dopaminergic neuronal cell death—complex 1 inhibition ↑ Mitochondrial fission and ↓ mitochondrial fusion—impairing MFN2 function Impaired autolysosomal function in ECGs	[80]
Maneb	SH-SY5Y cells Human A53T α-synuclein transgenic mice	↓ Cell viability Neurotransmitter reduction Neurotransmitter signaling alteration Dopaminergic neuronal cell death AEP inhibitor CP11-mitigated maneb-induced neurotoxicity	[81]
Deltamethrin	Wistar rats	↑ SOD activity ↑ Glutathione S-transferase activity ↑ MDA acid level ↓ Mitochondrial respiration—in the striatum and hippocampus	[82]
Fipronil	Sprague–Dawley Rat	Neurotoxic effect on nigrostriatal dopaminergic neurons	[83]

NADPH: Nicotinamide Adenine Dinucleotide Phosphate; PD: Parkinson’s disease; TH: Tyrosine hydroxylase; HSP 60: Heat Shock Protein 60; TLR: Toll-Like Receptors; MFN 2 Mitofusion 2; AEP: asparagine endopeptidase; NPCs: Neural Progenitor Cells; ECGs: Enteric Glial Cells; SOD: Superoxide Dismutase; MDA: Malondialdehyde; (−): inhibit; ↑ Inceased; ↓ Decreased.

Neurodegeneration occurs due to the oligomerization of amyloid proteins. Researchers suggest that oligomers can be triggered by intestinal bacteria. Substantial therapeutic benefits can be achieved by preserving a healthy microbiome to sustain the integrity of the intestinal barrier [13]. Gut dysbiosis, a key contributor to several gastrointestinal illnesses, may elevate lipopolysaccharides, pro-inflammatory cytokines, T helper cells, and monocytes, resulting in enhanced intestinal and blood–brain barrier permeability through the microbiota–gut–brain axis. Consequently, an accumulation of misfolded proteins, axonal damage, and neuronal demyelination occurs, hence promoting the development of neurodegenerative illnesses such as PD [84].

Pesticide exposure is increasingly recognized as an environmental trigger for PD through its disruptive effects on the gut microbiome [85]. In mouse models exposed to rotenone, gastrointestinal and motor dysfunctions were found to correlate with significant shifts in gut microbial composition. Commonly observed changes included increased abundance of genera such as *Lactobacillus*, *Bifidobacterium*, *Akkermansia*, and *Bacteroides*, alongside decreased levels of *Lachnospiraceae*, *Ruminococcaceae*, and *Clostridium*. These microbial alterations mirror patterns reported in human PD patients, particularly associations between increased *Lactobacillaceae* and *Bacteroides* and worsening clinical symptoms [85]. Furthermore, healthy mice that underwent gut microbiota transplantation from donors with PD exhibited motor dysfunctions in certain studies. These results collectively indicate that dysbiosis of gut microbiota is crucial in the etiology of PD. Nevertheless, the fundamental processes require more investigation [86]. Clinical trials and preclinical studies suggest that gut dysbiosis can impact PD progression by increasing intestinal permeability, aggravating neuroinflammation, aggregating abnormal α-synuclein fibrils, increasing oxidative stress, and decreasing neurotransmitter production [87].

Once established, pesticide-induced dysbiosis can set in motion a cascade of pathogenic events along the microbiota–gut–brain axis. An imbalanced microbiome often provokes local gastrointestinal inflammation and compromises the intestinal barrier, allowing bacteria and endotoxins to translocate into the circulation. This in turn triggers systemic immune activation, which has been linked to blood–brain barrier disruption, infiltration of peripheral immune cells into the central nervous system, and primed microglial activation [85].

Consequently, comprehending the impact of environmental variables, such as pesticides, on the gut, microbiota, and blood–brain barrier is essential for clarifying strategies for the prevention and treatment of gastrointestinal and neurological disorders [88].

Dardiotis et al. analyzed the blood concentrations of eight OCPs (hexachlorobenzene, heptachlor, heptachlor epoxide, c-chlordane, a-chlordane, p,p’-DDE, DDD, DDT) in 104 Greek PD patients and 110 healthy controls. All substances were detected in at least one sample, with p,p’-DDE and hexachlorobenzene being the most frequently found. Higher DDE levels were significantly associated with increased PD risk (OR = 2.592, 95% CI: 1.29–5.21), while lower HCB (hexachlorobenzene) levels were observed in PD patients (OR = 0.176, 95% CI: 0.09–0.35) [89]. A 2024 meta-analysis of 17 case–control studies revealed that exposure to OCPs elevates the incidence of PD, with a more pronounced connection in studies utilizing blood sample assessments (OR = 1.54) than in those employing indirect assessment methods (OR = 1.19). Additionally, OCPs were particularly correlated with an elevated risk of PD (OR = 1.18) [90].

### 4.2. Alzheimer’s Disease

AD is universally recognized as a complex illness resulting from the interplay of genetic and environmental variables. AD has a 70% risk attributed to genetic predisposition, with the remaining 30% risk related to environmental factors as well as individual lifestyle choices. These environmental variables encompass exposure to pesticides, toxic metals, air pollutants, and industrial chemicals. Prolonged exposure to these environmental contaminants and their bioaccumulation over a lifetime is believed to induce neuroinflammation and oxidative stress, promoting the development of neurodegenerative AD [91]. Moreover, research has revealed the effects of nutrition and exercise on the progression, prevention, and management of various disorders, including neurodegenerative diseases [92,93]. This perspective requires more exploration and validation through future investigations.

The key neuropathological characteristics are distinct neuronal degeneration, neuritic plaques, and neurofibrillary tangles (NFTs). Collectively, these alterations culminate in synaptic loss and neuronal cell death, leading to cognitive impairment. Moreover, chronic neuroinflammation is intricately linked to AD, exacerbated not only by the presence of Aβ (amyloid beta), tau, and neuronal destruction but also significantly intensifying these illnesses, resulting in additional harm. Nonetheless, the etiopathogenesis of AD remains incompletely understood, despite extensive discourse on the subject [94]. This disease is characterized by several symptoms, including cognitive impairment, environmental unresponsiveness, memory deficits, and challenges with thinking, language, and other cognitive functions [93,95]. Laboratory research has further elucidated the possible neurotoxic and neuropathological pathways caused by pesticides that may initiate or promote AD. The well-established pathological mechanisms encompass chronic oxidative stress, neuroinflammation, and Aβ/p-tau neuropathology, all of which adversely affect the aging neurons. In addition to these classical pathways, a number of emerging or less-characterized mechanisms have been proposed, such as somatic mutations, epigenetic modifications, impairment of adult neurogenesis, and dysbiosis of gut microbiota [96]. Studies indicate that the neurological alterations linked to AD may begin two decades or more prior to the manifestation of symptoms. Upon the onset of first changes, the brain adapts, allowing individuals to maintain normal functioning. As neuronal damage escalates, the brain loses its capacity to adapt, resulting in a gradual cognitive deterioration in individuals [97].

A cross-sectional study in Andalusia, Spain, analyzed 40,044 hospital records (2000–2021) to examine the link between pesticide exposure and AD. Using the Odds Ratio test, researchers found that districts with higher pesticide use had significantly higher AD prevalence and risk compared to lower-exposure areas [98]. Nonetheless, ecological studies are fundamentally constrained by their dependence on aggregated data and the incapacity to evaluate individual exposure levels. This constrains their ability to identify causal linkages and increases the risk of ecological error. Consequently, although these investigations are valuable for recognizing extensive patterns and formulating hypotheses, they require careful interpretation. To address these shortcomings and enhance the evidence base, there are biomonitoring-based studies that offer individual-level data. A study investigated OCP levels in the serum of 63 AD patients and 50 healthy controls, measuring oxidative stress markers—TAC, PCs, MDA, and NO—and enzyme activities—SOD, GPx (glutathione peroxidase), PON1 (paraoxonase 1), and AChE. The results showed significantly higher OCP levels in AD patients, while antioxidant enzyme activity and oxidative stress markers were significantly lower. Positive correlations between 2,4-DDE and MDA, as well as γ-HCH (Gamma-Hexachlorocyclohexane) and PCs, suggest that pesticide exposure contributes to AD development, likely through oxidative stress mechanisms linking environmental toxins to neurodegeneration [99]. Chlorpyrifos also induces oxidative stress and brain injury, leading to tau hyperphosphorylation at multiple AD-related sites. This occurs through the activation of glycogen synthase kinase-3β and inhibition of protein phosphatase-2A, causing a decline in hippocampal and cortical neurons. These pathological changes contribute to spatial learning and memory impairments, potentially progressing to AD [94]. Table 2 summarizes key studies investigating the neurotoxic effects of pesticide exposure, with a focus on their potential involvement in AD pathology.

## 5. Neurodevelopmental Disabilities

A revised literature study indicates that, from 2006 to 2014, the roster of acknowledged human neurotoxicants increased by 12 compounds, rising from 202 (including ethanol) to 214, averaging around two substances every year. A multitude of these compounds are extensively utilized and distributed across the worldwide ecosystem. Pesticides represent the predominant category among the newly discovered neurodevelopmental toxicants, consistent with the findings from 2006. During the same seven-year interval, the quantity of identified developmental neurotoxicants has increased from six to twelve. The rate of scientific discovery about novel neurodevelopmental risks is currently more accelerated than in previous times; yet, it remains slower than the detection of adult neurotoxicants [11].

The earliest stages of in utero development—characterized by rapid cellular proliferation, migration, and differentiation—are particularly vulnerable to environmental exposures. Toxicants encountered during this critical window can lead to developmental abnormalities, including neural tube defects. As fetal development progresses, continued exposure to harmful substances may interfere with essential neurological processes such as synaptogenesis, gliogenesis, myelination, and programmed cell death (apoptosis), potentially resulting in long-term functional and behavioral changes [14]. The fetus is notably underprotected against industrial chemicals. The placenta does not effectively block many environmental toxicants, allowing them to pass from maternal to fetal circulation. Additionally, some contaminants can be transmitted postnatally through breast milk, extending exposure into early infancy. During both fetal development and early postnatal life, the immature blood–brain barrier offers limited defense against the entry of these substances into the central nervous system, increasing the risk of neurodevelopmental disruption [11].

Postnatal neurodevelopment occurs in stages known as critical periods, during which brain circuits exhibit heightened sensitivity to environmental influences [104]. Prior research assessing the impact of low-dose chronic exposure to pesticide mixtures and other chemicals, designed to replicate real-life exposure scenarios, demonstrated that hormetic neurobehavioral effects may manifest after exposure to mixtures at doses deemed safe for individual substances. Furthermore, these effects can be intensified by the presence of specific conditions, such as vitamin deficiency [16]. A study monitored 4231 infants born in Pelotas, Brazil, evaluating their cognitive development at ages 2 and 4 with standardized assessments. At the age of 4, the prevalence of intellectual disability was 4.5%, with etiologies categorized as environmental (44.4%), genetic (20.5%), neonatal sequelae (13.2%), idiopathic (12.6%), and other disorders (9.3%). Although pesticide exposure was not directly assessed, these findings underscore the importance of early-life environmental influences on neurodevelopment and support the need for focused evaluation of neurotoxic exposures such as pesticides [105].

Autism spectrum disorder (ASD) and attention deficit disorder, with or without hyperactivity (ADHD), are prominent and thoroughly researched neurocognitive diseases in children. Other neurocognitive illnesses of interest, including learning disability, conduct disorder, intellectual disability, cerebral palsy, and sensory impairments in vision and hearing, sometimes exhibit similar neurocognitive symptoms or diagnoses [14]. Numerous diseases lack recognized biomarkers, have limited or no medical therapies, and can solely be identified by behavioral assessment [106]. The complex characteristics of ASD and ADHD have rendered it impractical to do mechanistic research on human volunteers. Similarly, human dosage trials have been deemed unethical. This is especially applicable to research in children. The development processes of the nervous system of small animals and humans are basically analogous, but they occur at varying speeds across the two groups. This facilitates the correlation of information from in vitro and animal research with findings from human epidemiological investigations [14]. Investigations into the etiology of ASD and ADHD, as well as their correlation with pesticide exposure, have advanced through several interconnected paths. Numerous cross-sectional and longitudinal epidemiological studies involving pesticide-exposed individuals have been conducted to identify various brain injuries [14]. Evidence suggests that maternal chemical intolerance—defined as a heightened reactivity to low-level chemical exposures—may be associated with an increased risk of ADHD and ASD in offspring. This connection presumably indicates heightened maternal exposure to environmental toxicants in conjunction with individual susceptibility variables, rather than solely maternal sensitivity. Heilbrun et al. argue that developmental toxicity is determined by the relationship between exposure dose and host sensitivity [15].

Recent epidemiological and longitudinal research indicates that prenatal exposure to pyrethroid insecticides in the environment is associated with an increased risk of autism, developmental delays, and developmental disorders overall. Prolonged exposure to neonicotinoids in humans was also associated with autism, cognitive impairment, and digit tremors [2]. Data analysis from the CHARGE research indicated a substantial elevation in the incidence of either ASD or developmental delay associated with prenatal exposure to pyrethroid pesticides administered within 1.5 km of the residence. Similarly, research in New York showed a correlation between regions utilizing aerial application of pyrethroid pesticides and the prevalence of ASD and developmental delays in those locations. Moreover, the detection of pyrethroid metabolites in blood or urine is associated with an increased risk of ADHD in children [107]. Although the MARBLES (Markers of Autism Risk in Babies—Learning Early Signs) study found a link between higher prenatal DAP metabolite levels and increased ASD risk in girls, this association was not observed in boys. Similarly, a case–control study in California showed that children of mothers living within 500 m of organochlorine pesticide-treated fields during pregnancy were six times more likely to develop ASD. Nevertheless, a Finnish study further emphasized that elevated maternal p,p′-DDE levels were associated with ASD, with an even stronger link found in cases involving intellectual disability [14]. Moreover, elevated maternal levels of p,p′-DDE were linked to an increased chance of premature birth in a study with a substantial sample size, and deficits in psychomotor development indices and other cognitive functions, along with delayed processing speed, were noted in the exposed children [108].

CHAMACOS study included 600 pregnant women, predominantly workers from agricultural families in the greatest agricultural area in the U.S., where the entire populace is extensively exposed to pesticides. Approximately 220,000 biological and environmental specimens, including blood, urine, and home dust, have been collected and are now preserved in a sample bank for future study purposes. The concentrations of OPP residues in maternal urine were quantified, revealing a correlation between these pesticide levels and the cognitive development of their children. The findings were highly significant: abnormal reflexes were seen in newborns, intellectual impairment was present in 2-year-olds, a decline in IQ was detected in 7- and 10-year-olds, and there was an increase in ADHD and ASDs [15].

Spatial learning and memory issues are well-documented effects of OPP intoxication, with consequent neuronal death in central nervous system regions [30]. Multiple epidemiological studies have investigated the association between prenatal exposure to OPPs and cognitive and behavioral development in children. These investigations frequently utilize neurodevelopmental screening instruments provided to children at different ages, with the objective of evaluating individual developmental performance [47]. A recently published study investigated the correlation between prenatal exposure to OPPs and neurotoxicity biomarkers in cord blood from 398 mother–child pairs within the GENEIDA birth group in Spain. Elevated maternal DAP metabolite levels, especially dimethyl groups, were associated with higher GFAP (glial fibrillary acidic protein) levels, whereas OPFR (organophosphate flame retardant) metabolites were connected with heightened GFAP (glial fibrillary acidic protein) and UCHL1 (ubiquitin C-terminal hydrolase L1), suggesting possible neurotoxic effects. Sex-specific differences were noted, with OPFRs correlated with elevated GFAP levels in boys, whereas diethyl DAP metabolites were connected with heightened BDNF (brain-derived neurotrophic factor) levels in girls [47]. Another study found that children whose mothers lived near high levels of OPPs and carbamate pesticide use during pregnancy had significantly lower IQ scores, particularly in Full-Scale IQ, perceptual reasoning, and working memory [109]. These findings align with other studies assessing OPP toxicity, which similarly report neurodevelopmental impairments associated with early-life exposure, reinforcing concerns about the lasting impact of these compounds on cognitive function. A study of 305 children aged 6–11 in agricultural regions of South-Eastern Spain found that higher exposure to OPPs is correlated with diminished IQ and verbal comprehension scores, particularly pronounced in boys. Postnatal exposure had the most robust correlations with cognitive impairments, but prenatal exposure exhibited a diminished effect, indicating that continuous pesticide exposure may substantially influence children’s neurodevelopment [110].

Magnetic resonance imaging (MRI) is a sensitive technique for illustrating in vivo changes in brain structure and function following acute OPP poisoning. Central nervous system effects of OPPs include nonspecific symptoms, such as irritability, agitation, disorientation, and confusion, which can evolve into generalized seizures and status epilepticus [30]. Shrot et al. evaluated the implications of paraoxon-induced neurotoxicity in rats, assessing cerebral alterations by MRI and spectroscopy at 3 and 24 h post-exposure. All animals poisoned with paraoxon had general convulsions, occurring within minutes of paraoxon administration. Brain edema was the most pronounced on MRI three hours post-poisoning. Brain metabolic dysfunction, shown by reduced N-acetyl-aspartate/total creatine ratios, was observed in all poisoned animals as early as 3 h post-exposure and persisted at lower levels compared to non-poisoned animals even 24 h after poisoning. Substantial associations were identified between imaging results (brain edema and spectroscopic alterations) and clinical outcomes (impaired learning, weight loss, and pathology score) [30]. A different study assessed the correlation between exposure to chlorpyrifos and brain structure by MRI in 40 children aged 5.9 to 11.2 years. They compared 20 children with high exposure to 20 children with minimal exposure for the variables influencing the surface of the cortex. All subjects had little exposure to ambient tobacco smoke and polycyclic aromatic hydrocarbons. Children with the highest exposure had a decrease in IQ and demonstrated structural alterations in brain areas related to attention and behavioral regulation. Frontal and parietal cortical thinning was seen, along with an inverse dose–response relationship between chlorpyrifos and cortical thickness [111].

Prenatal exposure to chlorpyrifos was assessed in umbilical cord blood from a cohort of 263 inner-city minority children, who were monitored prospectively. Children with prenatal chlorpyrifos exposure in the higher quartile (n = 43) were more likely than their peers to display mild or mild to severe tremors in one arm, both arms, the dominant arm, and the non-dominant arm. Logistic regression studies revealed substantial chlorpyrifos effects on tremors in both arms, including the dominant and non-dominant arm [112]. In minimizing OPP usage, the toxicological implications of alternative compounds necessitate careful examination. Pyrethroid pesticides have replaced OPPs as the predominant category of insecticides in residential pest control products; however, recent animal laboratory investigations and epidemiological studies indicate that prenatal exposure to pyrethroid pesticides may elevate the risk of detrimental neurodevelopment, behavioral issues, and negative emotional states [113]. Data from a population-based cohort study of older adults in Greece indicated that individuals without dementia residing near sprayed fields exhibited inferior neuropsychological performance, particularly in language, executive, and visual–spatial functioning, and attention [16].

Neonicotinoids are structurally categorized into chloropyridinyl, chlorothiazolylmethyl, and tetrahydrofuranylmethyl subclasses, with chloropyridinyl compounds—such as imidacloprid, acetamiprid, and nitenpyram—being the most widely used [114]. Numerous studies have been undertaken to evaluate the possible embryotoxic consequences associated with the administration of neonicotinoids [56]. These substances are metabolized in both humans and the environment into 6-chloronicotinic acid (6-ClNA), a compound increasingly recognized for its neurotoxic potential [114]. Recent evidence also shows that 6-ClNA crosses both the placental and blood–brain barriers, raising further concerns about its possible contribution to neurodevelopmental disruption [115]. Notably, 6-ClNA may exert stronger non-covalent interactions with target molecules than its parent compounds, potentially enhancing its neurotoxicity [114]. Despite the Environmental Protection Agency categorizing neonicotinoid insecticides as Class II (moderate) and Class III (slight) hazardous chemicals, current research indicates they may represent a major risk to human nervous system development [116].

Experimental animal studies have corroborated the effects of early-life pesticide exposure on neurodevelopmental outcomes, consistent with the varied findings in human research. Additionally, several in vivo and in vitro studies examine the cognitive effects of pesticides (Table 3). Zebrafish have emerged as a valuable and supplementary vertebrate model for investigating pesticide-induced neurotoxicity. These fish have also been utilized as an experimental model to evaluate pyrethroid-induced developmental neurotoxicity. Analogous to findings in mammals, exposure to pyrethroids in zebrafish induces behavioral disturbances, notably hyperactivity, especially with deltamethrin [31].

## 6. Conclusions and Perspectives

Concurrently, agriculture requires enhanced support to adopt a systems approach that reduces the use of neurotoxic pesticides while ensuring the availability of healthy food and economic viability for farmers. Considering the rising incidence of these conditions, developing and advocating these guidelines must be prioritized. Ongoing research is essential to comprehending the potential exposure risks, especially concerning neurotoxicity.

A further gap in research on the consequences of pesticide exposure is the vulnerability of the general population to intoxication. In most instances, studies examine farmers and control groups mostly consisting of the general populace. Nevertheless, few investigations included individuals indirectly exposed to pesticides and noted a level of cognitive damage. Consequently, in addition to the understanding of pesticide contamination in water, soil, air, and food, experts propose that the general populace is also vulnerable to exposure and negative consequences.

Research studies investigating health impacts from pesticide exposure generally concentrate solely on the active chemical, skipping the pesticide formulations. Research involving humans suggests that the assessment of toxicity should prioritize health-related outcomes above precise indicators of toxicity, particularly concerning early-life exposure to chemicals.

In conclusion, continued investigation into the neurotoxic potential of pesticide residues is vital—not only to deepen our scientific understanding but also to inform public health strategies, regulatory policies, and sustainable farming practices aimed at protecting both human health and the environment.

## Figures and Tables

**Table 2:** Pesticide-related neurotoxic effects contributing to AD.

Pesticide	Experimental Model	Dosage	Outcome	Reference
Malathion	Rats	100 mg/kg	Spatial memory deficits Apoptosis in the hippocampus tau hyperphosphorylation **↓** PSD93 levels ↑ TNF-α and IL-6 levels (−) PP2A Activated GSK-3β	[100]
Thiacloprid	Chicken embryos (Total = 780 eggs)	0.1–100 µg/egg	↑ Mortality and abnormalities—dose-dependent ↓ Levels of monoamines and amino acid neurotransmitters ↓ Activities of AChE and Na^+^/K^+^-ATPase ↓ Activity of CAT and SOD Downregulated mRNA expression	[101]
Diazonin	Rats (*n* = 7)	2 mg/kg	↓ APP ↓ APL2 ↑ TNF-α levels in the PFC—5 days post exposure	[102]
Chlorpyrifos	Rats (Total = 24)	16.324 mg/kg	↓ Dopamine ↓ Serotonin ↑ MDA ↑ NO ↓ MAO-A	[27]
Deltamethrin	Wistar rats	3 mg/kg/every 3 days, gavage	↑ Anxiety along with altered cellular adhesion and vasculature development evaluated at 6 and 12 months of age	[103]

↑ Increased; ↓ Decreased; (-) Inhibition; GSK-3β: Glycogen Synthase Kinase-3 beta; PP2A: Protein Phosphatase-2A; PSD93: Postsynaptic density 93; (−): inhibition; AChE: Acetylcholinesterase; CAT: Catalase; SOD: superoxide dismutase; APP: Amyloid Precursor Protein; APL2: Amyloid Precursor-Like Protein 2; TNF-α: tumor necrosis factor-α; PFC: prefrontal cortex; MAO-A: monoamine oxidase-A; MDA: malondialdehyde; NO: nitric oxide.

**Table 3:** In vivo and in vitro evidence of pesticide-associated cognitive and neurodevelopmental disorders.

Pesticide	Experimental Model	Outcome	Reference
Clothianidin + Thiacloprid Mixture	Rats (*n* = 10)	Histological changes in the brain—loss of tissue architecture, nucleosomal retraction, ↑ Pycnosis in granular neurons Short-term memory deficits Abnormal CNS activity	[2]
IM + AC + THM	Human neuroblastoma—SK-N-SH Lepidopteran (Sf-9) cells	IM + AC, IM + TH, AC + TH, IM + AC + TH—synergistic effects at <50 mg/L IM + AC and IM + TH—antagonistic effects at higher concentrations IM + AC—antagonism at >0.5 mg/L	[117]
IM DIC ATZ	Zebrafish (Total = 90)	IMD + DIC + ATZ → highest LPO Antioxidant enzyme activities (superoxide dismutase, catalase, and glutathione peroxidase)—significantly elevated in the CMD group	[118]
IM	SH-SY5Y cells	↓ Cell viability (chronic exposure) ↓ Neurite outgrowth, dose-dependent retraction during differentiation Potential oxidative stress	[116]
IM	iPSC	↓ Viability of human iPSC-derived neurons and microglia ↑ Synaptophysin = abnormal synaptogenesis Impaired neurogenesis Cell-type-specific toxicity	[119]
Acetamiprid	Neonatal Wistar Rats	Glutamate dysregulation Microglial activation Motor impairment Purkinje cell abnormalities	[120]
Cypermethrin	Rats	Neurodegeneration after 12 weeks Postnatal exposure effect Neurochemical alterations	[121]
Deltamethrin	Mice	↑ DAT Hyperactivity Deficits of working memory and attention Impulsive-like behavior	[122]
Deltamethrin	Mice	↑ Repetitive behaviors ↑ DAT protein levels ↑ Total striatal dopamine and dopamine metabolites	[107]
Deltamethrin	Rats (Total = 19)	Cognitive impairment ↑ Locomotor activity ↓ TH immunoreactivity	[55]
Deltamethrin	Rats (Total = 19)	Memory and emotional impairment ↓ TH immunoreactivity in SNpc and VTA ↑ TH immunoreactivity in dorsal striatum	[123]
Diazinon	Rats (*n* = 7)	Decline in inhibitory avoidance memory performance ↓ APP and APLP2 gene expression	[102]
Diazinon	Rats	Locomotor hyperactivity Region-specific enhancement in dopamine utilization Deficient attentional accuracy	[124]
Fenobucarb	Zebrafish	Caspase 3 and 9 activation ↑ ROS production Axon degeneration Central nerve and peripheral motor neuron damage	[125]
Chlorpyrifos	Mice	↓ Neuron and glia counts in anterior cingulate, prelimbic, and infralimbic areas of mPFC	[126]

↑ Increased; ↓Decreased; IM: Imidacloprid; AC: Acetamiprid; CNS Central Nervous System; THM: Thiamethoxam; DIC: Dichlorvos; ATZ: Atrazine; LPO: lipid peroxidation; CMD: combination exposure; iPSC: human-induced pluripotent stem cells; DAT: dopamine transporter; APP: Amyloid Precursor Protein; APLP2: Amyloid Precursor-Like Protein 2; ROS: Reactive Oxygen Species; TH: Tyrosine Hydroxylase; SNpc: substantia nigra pars compacta; VTA: ventral tegmental area; mPFC: medial prefrontal cortex.

## Data Availability

The data presented in this study are available through the RASFF (Rapid Alert System for Food and Feed) public dashboard, accessible at https://webgate.ec.europa.eu/rasff-window/portal/ (accessed on 27 May 2025). These data were obtained from publicly available sources within the public domain.

## Abbreviations

The following abbreviations are used in this manuscript:

RASFF	Rapid Alert System for Food and Feed
AD	Alzheimer’s Disease
PD	Parkinson’s Disease
MRLs	Maximum Residue Limits
HBGVs	Health-Based Guidance Values
ADI	Acceptable Daily Intake
HBM	Human Biomonitoring
UL	Upper Intake Level
GAPs	Good Agricultural Practices
AChE	Acetylcholinesterase
OPPs	Organophosphate Pesticides
OCPs	Organochlorine Pesticides
IL-1 α	Interleukin-1 alpha
IL-1 β	Interleukin-1 beta
IL-6	Interleukin-6
TNF-α	Tumor Necrosis Factor-α
DDT	Dichlorodiphenyltrichloroethane
VGSCs	Voltage-Gated Sodium Channels
GABAA	Gamma-Aminobutyric Acid
NAChRs	Nicotinic Acetylcholine Receptors
TAC	Total Antioxidant Capacity
MDA	Malondialdehyde
PCs	Protein Carbonyls
ROS	Reactive Oxygen Species
DNA	Deoxyribonucleic Acid
MPP+	1-Methyl-4-phenylpyridinium
JNK	c-Jun N-Terminal Kinase
NO	Nitric Oxide
SOD	Superoxide Dismutase
GST	Glutathione-S-Transferase
CpGs	Cytosine–Guanine Sites
SNP	Single-Nucleotide Polymorphisms
DMPs	Differentially Methylated Sites
PEX19	Peroxisomal Prostaglandin D2 Synthase Biogenesis Factor 19
WNT16	Wingless-Type Integration Site Family Member 16
ENTPD8	Ectonucleoside Triphosphate Diphosphohydrolase 8
DOPAL	3,4-Dihydroxyphenylacetaldehyde
NADPH	Nicotinamide Adenine Dinucleotide Phosphate
HSP60	Heat Shock Protein 60
TLR4	Toll-Like Receptor 4
AEP	Asparagine Endopeptidase
Akt	Serine/Threonine Protein Kinase
NPCs	Neural Progenitor Cells
EGCs	Enteric Glial Cells
HCB	Hexachlorobenzene
NFTs	Neurofibrillary Tangles
Aβ	Amyloid beta
GPx	Glutathione Peroxidase
PON1	Paraoxonase 1
HCH	Gamma-Hexachlorocyclohexane
PSD93	Postsynaptic Density 93
PP2A	Protein Phosphatase-2A
GSK-3β	Glycogen Synthase Kinase-3 beta
CAT	Catalase
APP	Amyloid Precursor Protein
APL2	Amyloid Precursor-Like Protein 2
PFC	Prefrontal Cortex
MAO-A	Monoamine Oxidase-A
ASD	Autism Spectrum Disorder
ADHD	Attention Deficit Hyperactivity Disorder
IQ	Intelligence Quotient
DAP	Dialkyl Phosphate
GFAP	Glial Fibrillary Acidic Protein
OPFRs	Organophosphate Flame Retardants
UCHL1	Ubiquitin C-Terminal Hydrolase L1
BDNF	Brain-Derived Neurotrophic Factor
MRI	Magnetic Resonance Imaging
6-CINA	6-Chloronicotinic acid
IM	Imidacloprid
AC	Acetamiprid
TH	Thiamethoxam
DIC	Dichlorvos
ATZ	Atrazine
LPO	Lipid Peroxidation
CMD	Combination Exposure
DAT	Dopamine Transporter
TH	Tyrosine Hydroxylase
SNpc	Substantia Nigra Pars Compacta
VTA	Ventral Tegmental Area
